# Intra-operative fractures in primary total knee arthroplasty - a systematic review

**DOI:** 10.1186/s43019-020-00054-3

**Published:** 2020-08-10

**Authors:** Prabhudev Prasad Purudappa, Sruthi Priyavadhana Ramanan, Sujit Kumar Tripathy, Sushrruti Varatharaj, Varatharaj Mounasamy, Senthil Nathan Sambandam

**Affiliations:** 1 Boston VA Medical Center, 150 S Huntington Avenue, Boston, MA 02130 USA; 2 Chennai, India; 3 Department of Orthopaedics, AIIMS, Bhubaneswar, 751019 India; 4 Burrell College Of Osteopathic Medicine, 3336 Balcones Dr, Irving, TX 75063 USA; 5 Dallas VA Medical Center, 4500 S Lancaster Road, Dallas, TX 75216 USA

**Keywords:** Intra-operative fracture, Distal femoral fracture, Tibial fracture, Patella fracture, Primary total knee arthroplasty

## Abstract

**Background:**

One of the rare complications of primary total knee arthroplasty is intra-operative fracture. Intra-operative fracture during revision knee arthroplasty has been well-documented but there is limited literature on fractures occurring during primary knee arthroplasty. We conducted a systematic review of the literature to compare and contrast the various studies to clearly define the predisposing factors, incidence, and characteristics of the fracture itself and to arrive at a consensus on the management and prevention of intra-operative fractures during primary knee arthroplasty.

**Methods:**

The PubMed/Medline, Cochrane, Scopus and Embase databases were searched using keywords “intra-operative fracture”, “distal femoral fracture”, “tibial fracture”, “patella fracture” and “primary total knee arthroplasty”. A total of 158 articles were retrieved and after further filtration and exclusion processing, 10 articles that evaluated intra-operative fractures in primary total knee arthroplasty were included for the review.

**Results:**

The reported incidence of intra-operative fractures varied from 0.2% to 4.4%. A higher incidence in female patients with a male to female ratio of 0.4 was reported. Posterior stabilized (PS) total knee arthroplasty was associated with higher risk of intra-operative femoral fractures by many authors in this review. Timing of occurrence and location of the intra-operative fractures can vary widely, with femoral fractures occurring more commonly during bone preparation, trialing and impaction of the final implant and tibial fractures occurring during preparation for the tibial keel and impaction of the tibial component.

**Conclusions:**

Intra-operative fractures during primary total knee arthroplasty are rare with higher risk associated with osteoporosis, rheumatoid arthritis, advanced age, female gender, chronic steroid use, metabolic bone disorders, PS type of femoral implant and difficult surgical exposure of the knee joint due to severe deformities. A plethora of management options have been utilized according to surgeon preference. Standard principles of fracture fixation and arthroplasty principles should be followed to achieve stable internal fixation and any unstable fracture site should be bypassed with the utilization of stemmed components. Satisfactory radiographic and functional outcome can be expected with appropriate treatment.

## Introduction

Total knee arthroplasty (TKA) is one of the most commonly performed surgeries to reduce the pain and disability associated with end-stage knee osteoarthritis. It has been estimated that by the year 2030 there will be a need for 3.48 million TKAs annually [1]. One of the rare complications of primary total knee arthroplasty is intra-operative fracture, with a prevalence of 0.39–2.2% [2, 3]. These fractures can occur at various stages of the procedure including surgical exposure of the knee joint, during bone preparation and during trialing and placement of the final components [2]. Several authors have reported on the incidence, risk factors, location, intra-operative and post-operative management and the outcome of intra-operative iatrogenic fractures [2–8]. Various risk factors for intra-operative fractures have also been identified including advanced age, osteoporosis, rheumatoid arthritis, chronic steroid use, female gender, metabolic bone disease and posterior stabilized arthroplasty [2–5]. Different treatment modalities have been described for the treatment of these fractures including internal fixation with screws, plating, tension-band wiring, use of stemmed components and augments with or without constrained implants and conservative methods including protected weight bearing with bracing [2, 3, 9–11]. We conducted a systematic review of the literature to compare and contrast the various studies reporting on intra-operative fractures associated with primary total knee arthroplasty, to clearly define the predisposing factors, incidence and characteristics of the fracture itself, and to arrive at a consensus on the management and prevention of intra-operative fractures.

## Material and methods

**Fig. 1:**
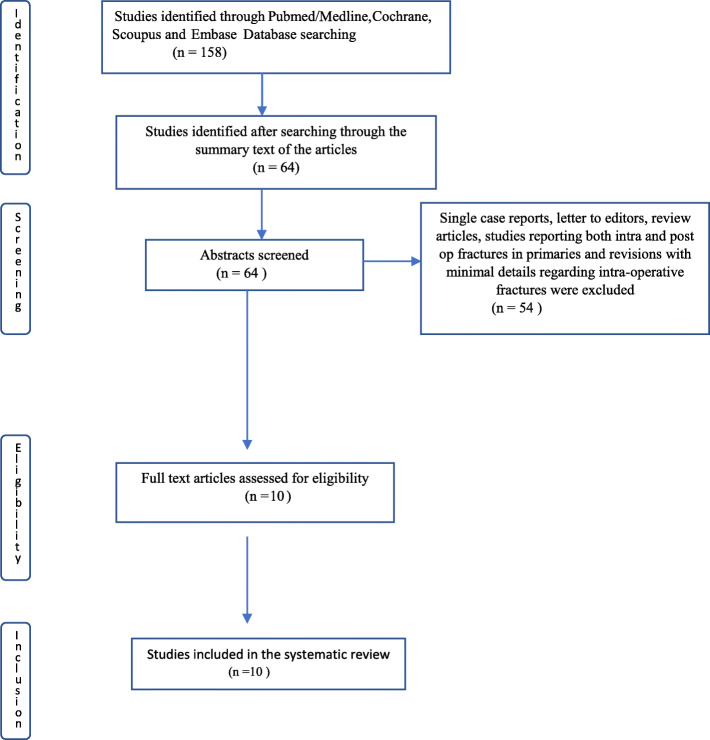
Preferred reporting items for systematic reviews and meta-analyses (PRISMA) flow chart

The PubMed/Medline, Cochrane, Scopus and Embase databases were searched using the keywords “intra-operative fracture”, “distal femoral fracture”, tibial fracture”, “patella fracture” and “primary total knee arthroplasty” to retrieve articles evaluating the outcome of intra-operative fractures in primary total knee arthroplasty (Fig. 1; Preferred reporting items for systematic reviews and meta-analyses (PRISMA) flow chart). A total of 158 articles were retrieved; after further filtration by searching through the summary text of the articles we identified 26 articles published in English. Single case reports, letters to the editor, review articles and studies reporting both intra-operative and post-operative fractures in primary and revision surgery and articles with minimal details of intra-operative fractures were excluded from this review. The abstracts of the remaining articles were read and only those articles that evaluate intra-operative fractures in primary total knee arthroplasty were included for the review. The references of these articles were also hand searched for any missing articles. A total of 10 articles were included in this systematic review.

## Results

### Patient demographics and incidence of intra-operative fractures in primary TKA

The incidence of intra-operative fractures reported in the literature varied from 0.2% [12] to 4.4% [13]. Even though Huang et al. [6] and Felix et al. [9] reported incidence of 0.04% and 0.07% respectively, these percentages do not represent the true incidence, because one study included only femoral condyle fractures and the other study included only tibial fractures (Table 1). The incidence of reported intra-operative fractures may be underestimated because clinically insignificant fractures may be missed [7]. Six reports [2, 4–7, 13] include details of the numbers of male and female patients; 39 were men and 112 were women, with a ratio of 0.34. Pinaroli et al. [3] report a male to female ratio of 0.38 and Felix et al. [9] report a ratio of 0.4. Delasotta et al. [7] found that intra-operative fractures are 4.44 times more likely to occur in women than in men.

**Table 1:** Patient demographics

Study	Mean age years	Gender Male (M) Female (F)	Mean follow up in months	BMI kg/m^**2**^	Pre-operative diagnosis	Incidence Number of fractures/total TKAs
**Alden et al.** [2]	65.2 ± 16 years	M^−12^ F^−54^	61 (2–184)	29.25 ± 6.08 (range, 17.59–49.87	Primary OA-57.4% Post-traumatic arthritis-11.8%, Rheumatoid arthritis (RA)-11.8%	0.39% 67/17,389
**Agarwala et al.** [4]	68.9 years.	M^− 7^ F^− 12^	28.7 (6–73)	NR	Primary OA-14 (73.6%) RA-4 (21.2%) post-traumatic arthritis-1	0.6% 19/3168
**Pinaroli et al.** [3]	71 ± 8 (20–95) years.	M:F-0.38	36.8 ± 34	NR	Primary OA-87% RA-7.1% Condyle necrosis-3.4%	2.2% (40/1795)
**Hernigou et al.** [8]	72(69–77) years	NR	NR	NR	NR	1.6% (10/617)
**Delasotta et al.** [7]	73.3 years	M^−1^ F^−5^	12.8 (2–39).	31.6 (23.7–50.1)	NR	0.4% (6/1469)
**Huang et al.** [6]	Case 1–49 Case 2–42	F Male	24	NR	RA RA	0.04% (2/5000)
**Pun et al.** [5]	71.6 years (54–83)	M^−5^ F^−12^	84.4	NR	NR	1.3% (17/1346)
**Lombardi et al.** [13]	IB-II PS – 70 Maxim PS-68	M^−13^ F^−28^	NR	NR	OA-573 RA- 29 Avascular necrosis (AVN)-5 OA-404 RA-37 AVN-2	Insall-Burnstein-II PS knee-4.4% 40/898 Maxim PS knee-0.2% 1/532
**Berry et al.** [12]	NR	NR	NR	NR	NR	0.2% (36/16,906)
**Felix et al.** [9]	70 years	M:F-0.4	53(8–144)	NR	Most common diagnosis-OA and RA	0.07%

*BMI* body mass index, *TKA* total knee arthroplasty, *OA* osteoarthritis, *NR* not reported

The mean age of the patients in the reported studies is 60 years with a range of 45.5–73.3 years. The studies report a mean follow up of 43 months. The most common indication for total knee arthroplasty was primary osteoarthritis, comprising 57.4% of the population in the study of Alden et al. [2] and 87% of the population in the study of Pinaroli et al. [3]. Rheumatoid arthritis was the second most common indication among the studies, comprising 21% of the population reported by Agarwala et al. [4].

### Risk factors

Osteoporosis, rheumatoid arthritis, advanced age, female gender, chronic steroid use, posterior stabilized arthroplasty and metabolic bone disease have been reported by several authors to be significant risk factors for intra-operative fractures during primary total knee arthroplasty [2–6, 11–16]. As mentioned previously, many authors found a significantly higher incidence of intra-operative fractures in women as compared to men [2, 4–7, 9, 13]. Even though Lombardi et al. [13] identified a twofold difference between men and women with fracture (13 men, 28 women), the same male to female ratio was also observed in the Insall-Burnstein II posterior-stabilized (IB-II PS) nonfractured group, and the difference between men and women was not statistically significant.

Pinaroli et al. [3] utilized anterior tibial tuberosity elevation for the exposure in 137 out of the 1795 TKAs; 12 of them (8.7%) developed tibial fractures intra-operatively thus indicating significantly increased risk of intra-operative fractures in association with tibial tuberosity elevation. Delasotta et al. [7] reported that patients with severe varus or valgus deformity requiring a semi-constrained implant are at higher risk of intra-operative fracture, as more bone is resected and the box cut is larger. Both cases reported by Huang et al. [6] were in patients with rheumatoid arthritis and severe osteoporosis. One had severe valgus deformity with tibial bone defects and a pre-operative range of motion (ROM) of 90°. The other patient had a severe varus deformity with medial tibial bone defect and femoral bone defects. This patient also had a very limited ROM of 40° pre-operatively, thus making the surgical exposure difficult.

### Classifications

Felix el al [9]. classified periprosthetic fractures of the tibia into to types I– IV based on their location, and they were subdivided in to type A - post-operative fracture with a well-fixed prosthesis, type B - post-operative fracture with a loose prosthesis and type C - intra-operative fracture. The classification includes both intra-operative and post-operative fractures. Type I fractures extend from the tibial plateau and involve the prosthesis interface, type II fractures occur adjacent to the tibial stem in the proximal metaphyseal-diaphyseal region, type III fractures are distal to the tibial prosthesis and type IV fractures are limited to the tibial tubercle. We did not find any particular classification for intra-operative femoral fractures in primary knee arthroplasty.

### Type of implants and associated risk of intra-operative fractures

Alden et al. [2] reported that a relative risk of femoral fracture in PS TKA of 4.74 as compared to cruciate retaining (CR) knee surgery. The majority of the distal femoral fractures in their study involved the medial femoral condyle (30%) followed by the lateral femoral condyle (17%). Lombardi et al. [13] evaluated the incidence of intra-operative intercondylar fractures associated with two different type of implant designs. Among the 898 IB-II PS (Zimmer Warsaw) knee implants, 40 were found to have the intra-operative intercondylar distal femoral fracture. After they started using Maxim PS knee (Biomet, Warsaw), they used a special instrument to size the intercondylar resection before the insertion of the final component. This allowed the surgeon to find out whether or not the intercondylar resection was adequate for seating the final component. They reported one displaced lateral condylar fracture out of the 532 Maxim PS TKAs. The difference in the incidence of intercondylar fractures between these two cohorts of patients was statistically significant. Hernigou et al. [8] also reported that 6 of the 10 intra-operative femoral fractures were associated with PS knee surgery and they involved the medial condyles. Delasotta et al. [7] reported intra-operative fracture rates of 0.32%, 0% and 3.13% in PS, CR and semi-constrained implants, respectively. They also found that intra-operative fracture was 9.69 times more likely to occur in patients with a semi-constrained implant than in those with a PS implant.

Pinaroli et al. [3] found that the incidence of tibial fractures was higher with the use of smaller tibial components. Out of the six tibial sizes available from the manufacturer (Tornier PS knee), use of the size-1 tibia was associated with statistically significantly higher incidence of tibial fractures. They found that proportionally, the tibial keel for base plate size 1 was too large for the tibia of patients with a small frame, which explained the higher incidence of fracture with the size-1 tibial implant.

### Time of occurrence of the fractures during surgery

Pun et al. [5] reported that, of the femoral fractures identified (*n* = 5), 80% (*n* = 4) were avulsion fractures in the coronal plane of the medial femoral condyles, which occurred during removal of the intercondylar notch because of an incomplete sagittal cut between the intercondylar notch and medial femoral condyle. Agarwala et al. [4] reported that of the femoral fractures identified (*n* = 4), 75% (*n* = 3) occurred during removal of the intercondylar notch bone and trialing. The majority of fractures (39%) in the study of Alden et al. [2] occurred during exposure and bone preparation, 33% occurred during trialing of the components and another 19% occurred during cementation. Of the 10 non-displaced femoral condyle fractures reported by Pinaroli et al. [3], 9 were observed during impaction of the PS femoral implant. Delasotta et al. [7] reported that 50% of the femoral fractures occurred during trialing when the tibia was reduced on to the femur and another 50% occurred during final implantation.

Of the tibial fractures in the study of Agarwala et al. [4], 53% occurred during placement of the final cemented component. Overzealous hammering of the final tibial component has been recognized to be a strong risk factor for intra-operative tibial fractures [4, 10]. Of the tibial fractures identified by Pinaroli et al. [3] and by Pun et al. [5], 90% and 100%, respectively, occurred during preparation of the tibial keel or impaction of the final tibial implant.

### Location and characteristics of intra-operative fractures

There were 150 femoral fractures and 98 tibial fractures in total from the 10 studies included in this review. Of the 10 studies, 5 reported both femoral and tibial fractures [2–5, 12], 4 reported only femoral fractures [6–8, 13] and 1 study reported only tibial fractures [9]. Alden et al. [2] and Berry et al. [12] reported that the majority of fractures in their studies were femoral as compared to tibial fractures, whereas in three other studies the majority of fractures were tibial as compared to femoral fractures [3–5] (Table 2).

**Table 2:** Characteristics of the fractures and intra-operative details

Study	Location of the fracture		Displaced versus non-displaced	Type of implant used	Time of fracture occurrence	Method of fracture management
**Alden et al.** [2]	Femur−49 Tibia-18 Patella-0	Medial femoral condyle-20 Lateral femoral condyle-11 Supracondylar femur -8 Medial epicondyle-7 Lateral epicondyle-2 Posterior cortex-1 Lateral tibial plateau-6 Medial tibial plateau-3 Anterior cortex-4 Lateral cortex-3 Medial cortex-10 Posterior cortex-1	64 displaced 3 non-displaced were not detected intra-operatively	All cemented 48 posterior-stabilized (PS) 9 cruciate retaining (CR) 10 with additional constraints 1 distal femoral replacement	26 during exposure and preparation, 22 while trialing, 13 during cementation, and 3 while inserting the poly-ethylene spacer	10 were stable-treated non-operatively Screws, washers and plate fixation-29; stemmed femoral components-8; stemmed tibial components-8; screws, washers and stems-3; screws, washers, suture-2; suture-2; allograft strut-1; bone graft and cement-1; distal femoral replacement-1; screws and plate-1; stemmed constrained condylar knee component-1; and threaded Steinman pin-1
**Agarwala et al.** [4]	Femur-4 Tibia-15 Patella-0	2 medial condyle avulsion 1 lateral condyle 1 medial epicondyle 6 lateral tibial cortex 2 lateral tibial plateau 4 medial tibial plateau 3 anterior tibial cortex	All displaced	All cemented Posterior stabilized Genesis II Smith & Nephew Patella not resurfaced	6-during exposure and bone preparation; 4- during trialing of components; 8-during cementing and final implantation; 1-unknown time	2 medial and 1 lateral femoral condyle fractures with plate and screws; 1 medial epicondyle fracture with screw Lateral tibial cortex fractures-3 with screws and 3 with sutures; 4 medial tibial plateau fractures and 2 lateral tibial plateau fractures with stemmed tibial component; anterior tibial cortex fractures-1 with screw and 2 with sutures.
**Pinaroli et al.** [3]	Femur-10 Tibia-30 Patella-0	Metaphyseal femur-9 Anterior femoral cortex perforation from IM rod-1 Fissures of the tibial plateau-27 Multifragmented epiphyso-metaphysodiaphyseal tibial fracture-1 Tibial tuberosity fracture-2	Non-displaced Displaced Displaced	Cemented Tornier Posterior stabilized knee	During impaction of the femoral implant During intramedullary jig placement During preparation of the tibial keel or impaction of the tibial implant. During knee hyperflexion for tibial preparation after tuberosity elevation During screw fixation after elevation of tuberosity	Stable-delayed weight bearing for 1–2 months No intervention Long tibial keel with figure-of-eight wire fixation supported by two screws of 4.5 mm diameter on each side of the keel Long-stem tibia with plate fixation Screws reinforced by metallic wires
**Hernigou et al.** [8]	Femur-10	Medial condyle-9 Supracondylar-1	All displaced	Cemented PS knee	6 medial condyle fractures associated with PS knee-occurred while placement of the femoral component. 3 medial femoral condyle fractures occurred with a retractor on the posterior aspect of the tibia during tibial implantation Supracondylar fracture-occurred while trialing in a female patient with hip arthrodesis	Medial condyle fracture fixed with one or two transverse screws Femoral component with stem extension
**Delasota et al.**	Femur-6	Medial condyle-2 Lateral condyle-2 Intercondylar-2	NR	Cemented Stryker Triathlon 4-PS knees 2-semi-constrained	50% occurred during trialing, while tibia was reduced onto the femur 50% occurred during final implantation	NR
**Huang et al.** [6]	Case 1 Case 2	Medial femoral condyle Lateral femoral condyle	Displaced Displaced	Cemented PS total knee NRG, Stryker Cemented PS total knee, PFC, Depuy	During trailing of the femoral component, a medial femoral condylar fracture occurred Occurred at the time of trialing of the femoral component	Reduced and internally fixed with two 6.5 mm × 30 mm cancellous screws Reduction and internal fixation with two 4.5 mm × 30 mm cancellous screws
**Pun et al.** [5]	Femur-5 Tibia-12	Medial femoral condyle-4 Medial cortex of the medial condyle-1 Vertical crack fractures of the anterior cortex of the medial tibial plateau	Minimally displaced Minimally displaced	Cemented PS NexGen LPS fluted tibial	Medial femoral condyle avulsion fracture occurred during removal of the intercondylar notch Medial cortex of medial femoral condyle fracture- during removal of large osteophyte Tibial fractures occurred during hammering down of the final tibial component	Multiple 3.5-mm partially threaded AO cancellous screws Multiple 3.5-mm partially threaded AO cancellous screws
**Lombardi et al.** [13]	Femur-40 Femur-1	Intercondylar Lateral condyle	35 non-displaced 5 displaced 1 displaced	Insall-Burnstein-II posterior stabilized (Zimmer, Warsaw) Maxim posterior stabilized (Biomet, Warsaw)	Non-displaced fractures identified in post-operative radiographs Displaced fractures-timing not reported Angular removal of the trial	Non-displaced fractures identified post-operatively (post-op)-no change in post-op protocol 4 displaced-screw + stemmed component 1 displaced-screw only Screw only
**Berry et al.** [12]	Femur-23 Tibia-13	Most were intercondylar fractures that occur with placement of a PS knee implant-no other details	NR	NR	NR During tibial preparation, trial reduction, and implant placement	NR
**Felix et al.** [9]	Tibia-10	Type 1C-6 Type IIC-3 Type IIIC-1	NR	NR	Retraction on bone, tibia preparation, trial reduction, seating of the implant	Different techniques-screw fixation, K-wire fixation prior to cementing a stemmed tibia, filling the defect with cement

*NR* not reported, *PS* posterior-stabilized

Among the studies that reported details, the medial femoral condyle was the most common fracture site, followed by the lateral femoral condyle involving the distal femur [2, 4, 8]. Three studies reported femoral fractures as intercondylar or metaphyseal fractures, without further specification of the fracture site [3, 12, 13]. Alden et al. [2] reported 8 supracondylar fractures out of 49 femoral fractures and Hernigou et al. [8] reported 1 supracondylar fracture out of 10 femoral fractures. No other studies reported supracondylar fractures. Fractures of the medial and lateral femoral epicondyles were uncommon [2, 4].

Alden et al. [2] and Agrawala et al. [4] reported lateral and medial tibial plateau fractures, which are similar to the Type IC fracture described by Felix et al. [9]. The large majority of the reported tibial fractures involved the anterior, posterior, medial or lateral cortex of the tibia without significant displacement. Pinaroli et al. [3] reported one complex metaphyseo-diaphyseal tibial fracture, which was treated with a long-stem tibial implant along with plate and screw fixation. They also reported two fractures of the anterior tibial tuberosity.

### Management options

There is no consensus in the literature on a particular treatment option for a particular type of fracture. We found that various treatment options were utilized according to surgeon preference, including no fixation with or without delayed weight bearing in stable non-displaced fractures, screw fixation, sutures, figure-of-eight wire, components with intramedullary stems, plate and screws, constrained knee implants, distal femoral replacement and various combinations of these techniques.

Lombardi et al. [13] reported that 35 out of 40 femoral intercondylar fractures were non-displaced, and they were treated with no fixation and no change in the post-operative protocol, with full weight bearing and ROM as tolerated. Alden et al. [2] and Pinaroli et al. [3] delayed weight bearing in stable femoral fractures for 6 weeks. We found two studies in which screws and stemmed femoral components were used in some of the femoral condyle fractures [2, 13]. Three authors utilized only screws for the fixation of femoral condylar fractures [5, 6, 8]. Agarwala et al. [4] fixed the displaced medial and lateral femoral condylar fractures with plate and screws; similarly Alden et al. [2] fixed some femoral condylar fractures using plates and screws, but they did not provide details on which type of femoral condylar fractures were indicated for the use of plates and screws as compared to screws alone.

Vertical crack fractures of the tibial cortex were fixed with screws in four studies [2, 4, 5, 9]. Agarwala et al. [4] used a stemmed tibial component to fix 4 medial tibial plateau and 2 lateral tibial plateau fractures, and Alden et al. [2] used a stemmed tibial component in 8 out of the 18 tibial fractures in their study. Pinaroli et al. [3] treated 27 vertical fractures of the tibial plateau with a long tibial keel and Fig. 8 wire supported by two screws of 4.5 mm diameter on each side of the keel. Tibial tuberosity fractures were fixed with screws reinforced with metallic wires [3] (Table 2).

### Post-operative treatment protocol

There was variation among the studies in allowing weight bearing and ROM after the intra-operative fractures were identified. Non-weight bearing or partial weight bearing for a period of 6–8 weeks post-operatively was instituted in four studies [2, 3, 6, 9]. Full weight bearing and ROM with crutches or a walking frame immediately after surgery was allowed in three studies [4, 5, 13].

### Clinical and radiographic outcomes

Different outcome scores were utilized and all the authors reported significant improvement in the clinical scores as compared with the pre-operative status [2–7, 13]. Pinaroli et al. [3] reported an International Knee Society (IKS) score of 88.2 (43–100) at the final follow up and a function score of 72.8 (0–100) in patients with tibial fractures and an IKS score of 89.1 (60–100) at the final follow up and a function score of 75.6 (40–100) in patients with femoral fractures. There was no statistically significant difference in comparison to the series of patients without complications. Data on radiographic healing of the fractures was available in 7 [2–7, 13] out of the 10 studies and all of them reported radiographic healing of all fractures except for one complex fracture of the tibia reported by Pinaroli et al. [3], which went on to non-union and required multiple surgeries (Table 3).

**Table 3:** Post-operative outcome

Study	Revision surgery	Clinical outcome	Radiographic outcome	Post-operative physiotherapy protocol
**Alden et al.** [2]	14 of the 66 patients (21%) underwent revision arthroplasty at an average of 2.8 years Instability-4, loosening and osteolysis-4, infection-2, anterior knee pain-1, patellar mal-tracking-1, stiffness-1, progression of metastatic disease-1	Knee extension improved from 7.1° pre-operatively to 1.8° post-operatively. Knee flexion remained unchanged at 103°. Mean Knee Society score (KSS0 improved from 46.4 pre-operatively to 79.5 post-operatively. Mean KSS function score increased from 34.5 pre-operatively to 61 post-operatively	All fractures healed radiographically	Non-displaced fractures - touch weight bearing for 6 weeks
**Agarwala et al.** [4]	No patients required revision surgery over the follow up	The mean KSS pre-operatively was 35.7. Post-operatively, KSS at first follow up was 81 and AT last follow up was 92	All patients achieved bony union at a mean duration of 8.9 weeks (range 5–16 weeks)	Full range of motion of the operated knee with full weight bearing using a walker
**Pinaroli et al.** [3]	1 with tibial tuberosity fracture at 3 months-ORIF 1 patient with femorotibial instability-liner exchange at 23 months 1 patient with complex metaphyseo-diaphyseal fracture required multiple operations including revision fixation and bone grafting, two-stage revision for infection	Tibial fractures-IKS score-88.2 Function score-72.8 Femoral fractures IKS score-89.1 Function score-75.6	All fractures healed except one with complex tibia fracture which required multiple operations	Stable femoral fractures-delayed weight bearing for 1–2 months No report on tibial fractures
**Hernigou et al .** [8]	NR	NR	NR	NR
**Delasotta et al.** [7]	None during the reported follow up	Average range of motion-0 – 116° Knee Injury and Osteoarthritis Outcome score-76.8 (64.3–91.7) UCLA score-5 (4–6)	All fractures healed	NR
**Huang et al.** [6]	None	Case 1-Knee Society and function scores were 70 and 65, respectively, at 2-year follow up Case 2-Knee Society and function scores were 68 and 70, respectively, at 2-year follow up	Union confirmed at 3-month follow up Union confirmed at 3 month follow-up	Active and passive ROM exercises implemented. A brace was used and partial weight bearing with a walker encouraged for the first 2 post-operative months
**Pun et al.** [5]	One patient developed hematogenous infection secondary to an infected ingrown toenail and underwent revision TKA 9 months later.	Mean KSS-89.7 Function score-73.2	All patients achieved bone union	Patients were allowed full range of knee motion and full weight bearing with a walking frame on day 1. The walking frame was replaced by 2 crutches as patient mobility progressed. At 4 weeks, mobilization with one crutch or a single walking stick was allowed
**Lombardi et al.** [13]	None	IB-II PS knee-Pre-operative HSS score of average 55, average total postoperative score of 83 Maxim PS-average total pre-operative HSS score was 60, post-operative-81	All fractures healed	No change from the usual protocol. Full weight bearing and range of motion
**Berry et al.** [12]	NR	NR	NR	NR
**Felix et al.** [9]	1 required revision at 3 months with hinged prosthesis. Initial intra-operative fracture through a cyst was treated with curettage and cement, which failed with loosening of the tibia component	NR	NR	Non-weight bearing or partial weight bearing for 2 months

*NR* not reported, *ROM* range of motion, *TKA* total knee arthroplasty

### Revision surgery

Revision surgery was reported in 4 out of the 10 studies [2, 3, 5, 9]. Alden et al. [2] reported a revision rate of 21% (14 out of 66) at an average of 2.8 years (range of 2 months to 12 years), due to various reasons including instability (*n* = 4), loosening and osteolysis (*n* = 4), infection (*n* = 2), anterior knee pain in an un-resurfaced patella (*n* = 1), patellar mal-tracking (*n* = 1), stiffness (*n* = 1) and progression of metastatic disease (*n* = 1): in 12 (86%) of the knees requiring revision surgery, the intra-operative fractures were located in the distal femur and 2 fractures were located in the tibia [2]. Revision surgery rates of 7.5% [3], 6% [5] and 10% [9] were reported for the other three studies (Table 3).

## Discussion

Intra-operative fracture during primary total knee arthroplasty is uncommon and there is limited literature on this topic. We conducted a systematic review of the available literature to define the incidence, risk factors, time of occurrence of the fracture during surgery, characteristics of the fractures, management options and the outcomes. We have also identified precautions to be taken to prevent these fractures.

We identified incidence ranging from 0.2% to 4.4% but this may be underestimated as some of the intra-operative fractures may go un-noticed, and minimally displaced fractures not requiring any intervention may not be reported [7, 17]. The majority of the authors in this review reported incidence below 1% [2, 4, 6, 7, 9, 12]. In comparison, intra-operative fractures during revision knee arthroplasty have been reported to be 1.9–3% [12, 15]. Similar to revision knee arthroplasty surgery, osteoporosis, rheumatoid arthritis, advanced age, female gender, chronic steroid use, posterior-stabilized knee arthroplasty and metabolic bone disorders have been identified to be important risk factors for intra-operative fractures during primary knee arthroplasty [2, 4, 12, 13] and this review confirms these findings. Complex primary knee arthroplasty associated with severe pre-operative deformities, bone defects in patients with rheumatoid arthritis and osteoporosis are also important risk factors for intra-operative fractures [6]. Hernigou et al. [8] reported three patients with medial femoral condyle fractures that developed while using a counter angled retractor placed on the posterior aspect of the proximal tibia for cementing of the tibial implant, in patients with rheumatoid arthritis or in those in cases where it is difficult to expose the joint.

The intercondylar notch cut acts as a stress riser by decreasing the strength of the femoral condylar bone stock thus leading to fractures [18]. Many authors report significantly higher incidence of intra-operative femoral fractures associated with PS knee implants [2, 7, 13]. In a PS knee replacement, stresses delivered to the condyles from a tight-fitting final component into the intercondylar resection can result in a fracture [13]. Similarly, if the implant or the trial are inserted or extracted in a slight varus or valgus angulation results in stresses applied to either condyles, this may lead to fractures [13]. The distance from the medial cortex to the deepest aspect of the trochlea is smaller than that of the lateral femoral cortex to the bottom of the trochlea, and this predisposes the medial femoral condyle to higher risk of intra-operative fractures, especially with PS implants [8].

Bozkurt et al. [19] volumetrically measured and compared the amount of bone removed through the intercondylar femoral notch using implants from five different TKA manufacturers. They compared a single femoral size from each manufacturer. They found that the amount of bone removed from the intercondylar notch varied among the different manufactures, when comparing the Vanguard (3.6 ± 0.4 cm^3^), Nex-Gen (3.7 ± 0.5 cm^3^), Sigma (5.7 ± 0.4 cm^3^), Genesis 2 (6.3 ± 0.3 cm^3^) and Scorpio NRG (6.7 ± 0.7 cm^3^). Indelli et al. [18] compared the amount of intercondylar bone resection using three different PS TKA devices – the Sigma PS (De Puy, Johnson & Johnson, Warsaw, IN, USA), the Persona (Zimmer, Warsaw, IN, USA) and the Vanguard (Biomet Inc., Warsaw, IN, USA). They compared the bone removal area for each of the three groups of cutting jigs for small, medium and large sizes, through direct measurement with a millimeter caliper. They found that for all implant sizes the Zimmer Persona jig had a significantly inferior tridimensional box area resection compared to the Biomet Vanguard and Sigma PS. The difference between the Zimmer Persona and the Sigma PS was even more statistically significant in small and medium-sized implants. Their study demonstrates that, as the size of the femoral implant decreases, the amount of intercondylar bone resection also decreases significantly in some designs as compared to others. This review demonstrates that the PS knee design is an independent risk factor for intra-operative fractures during primary knee arthroplasty. Surgeons should exercise caution while using the PS knee design especially in female patients and in patients with osteoporosis or rheumatoid arthritis and those who require smaller implants.

Intra-operative fractures can occur at various stages of a knee arthroplasty procedure. Exposure and bone preparation in the femur have been identified to be particularly risky [2, 4] as is impaction of the final PS-type implants [3, 8, 13]. Huang et al. [6] were of the opinion that the microfractures that might have occurred during exposure and bone preparation would become visible on trialing of the implants due to the mechanical forces created by reducing the tibia on to the femur.

A high revision rate of 21% was reported by Alden et al. [2], which may have been due to many of these patients having previous knee surgery or severe bone deformity. Of the 14 patients who underwent revision TKA, 7 had prior knee surgery including femoral or tibial osteotomies: in addition, 2 patients with prior osteotomies had severe bony deformities secondary to diagnosis of underlying osteogenesis imperfecta and Marquio syndrome. They concluded that this is a subset of patients that is more susceptible to peri-operative complications and thus poor outcomes.

### Recommendations and principles of management of intra-operative fractures

We found that a particular treatment approach is difficult to recommend because of variable fracture patterns and use of multiple treatment options by the authors reviewed in this study. As a general rule, fracture fixation and arthroplasty principles should be followed to achieve stable internal fixation and any unstable fracture site should be bypassed with the utilization of long intramedullary stems [2, 4].

### Femoral fractures

Non-displaced intercondylar femoral fractures that do not extend into the medial or the lateral cortex are considered to be stable and they do not need any further intervention. Lombardi et al. [13] treated them with observation with no changes to the post-operative protocol. Full weight bearing and unrestricted range of motion were allowed.

Non-displaced fractures of the medial or the lateral femoral condyles extending into the respective cortices can be treated with partially threaded cancellous screws [5, 6, 8]. Huang et al. [6] were of the opinion that femoral condylar fractures can be successfully fixed using two cancellous screws, because perfect reduction of intra-operative fracture is easy to achieve, unlike post-operative fracture, and cemented fixation provides immediate stable fixation of the femoral implant. Moreover, plate fixation can interfere with the positioning of the femoral component. Plate and screw fixation [4] or screw with a stemmed femoral component [2, 13] should be available as options in displaced unstable femoral condylar fractures. Epicondylar fractures are uncommon and can be safely addressed using screw fixation [2, 4].

Highly comminuted femoral condylar fractures and supracondylar fractures might need to be treated using distal femoral replacement [2, 20].

### Tibial fractures

Small vertical crack fractures involving the anterior, posterior, medial or the lateral cortex of the proximal tibia can be managed with compression screws and a standard tibial implant [4, 5]. Tibial plateau fractures should be addressed using a stemmed tibial implant and compression screw fixation of the fracture to gain stable fixation [2–4, 9]. Fractures involving the metaphyseo-diaphyseal region of the tibia should be addressed using a stemmed tibial component with or without plate and screw fixation as needed [3, 9].

### Recommendations for prevention of intra-operative fractures

#### Pre-operative considerations

The factors implicated in increasing the risk of intra-operative fractures include osteoporosis, rheumatoid arthritis, advanced age, female gender, chronic steroid use, metabolic bone disorders such as osteomalacia, Paget’s disease, osteopetrosis, osteogenesis imperfecta and β-thalassemia [2, 4, 6, 9, 12, 15, 16]. Patients in whom one or more of these factors are identified warrant careful planning to prevent intra-operative fractures. Full medical history and careful evaluation of bone stock and bone mineral density testing should be undertaken. When indicated, patients with osteoporosis, rheumatoid arthritis, chronic steroid use and metabolic bone diseases should be evaluated by a medical specialist for appropriate pharmacological treatment to improve bone mineral density. Complete up-to-date imaging studies should be available at the time of surgery to make sure that the plan that was made in the clinic is still appropriate. Stemmed components, augments, plates and screws should be available as backup while performing primary knee arthroplasty in patients with any of the aforementioned risk factors.

Female patients are at particularly high risk of intra-operative femoral fractures due to their narrower distal femur. Using a PS knee implant along with excessive medial or lateral placement of the components weakens the respective condyles, thereby predisposing to fractures [4, 13]. A CR design or ultra-congruent polyethylene insert, which do not need accessory bone resection for the PS implant should be considered [18].

#### Exposure

The joint should be generously exposed to free the medial and lateral gutters before flexing the knee in patients with poor bone quality [2]. In patients with advanced arthritis with severe fixed varus and valgus deformities, adequate medial and lateral releases, respectively, should be performed prior to flexing the knee [2, 4, 6, 8].

#### Bone preparation, trialing and final component placement

Mismatch between the intercondylar resection and the final component should be avoided by carefully checking the appropriate preparation of the intercondylar notch in a posterior stabilized knee replacement using implant-specific trials [13]. In a PS knee design, the box cut should be wide enough to be slightly larger than the dimensions of the final implant box, to prevent initiation of the fracture during impaction [8]. Also the implant should be lateralized to increase the distance between the medial femoral cortex and the bottom of the box cut. Fractures of the femoral condyle during exposure of the proximal tibia can be prevented with improved exposure of the tibia obtained by disengaging the tibia forward before hyper-flexing the knee and externally rotating the tibia, and by avoiding placement of the posterior tibial retractor in patients with poor bone quality [8].

If any difficulty is encountered during removal of the intercondylar notch, a possible connection between the intercondylar notch and the femoral condyles from an incomplete box cut should be excluded to avoid an avulsion fracture [5]. Any varus or valgus malalignment during insertion and extraction of the trial and final femoral component should be avoided by using appropriate instrumentation rather than hand insertion [13].Overstuffing of the tibia with cement with overzealous hammering during final seating of the tibial component should be avoided [4]. Tibial tuberosity elevation is associated with 8.7% incidence of intra-operative tibial fractures [3]. Use of bone-holding forceps is recommended to decrease the risk of tibial fracture during impaction of the tibial base plate in smaller patients in whom tibial tuberosity elevation is necessary [3].

## Conclusions

Intra-operative fractures during primary total knee arthroplasty are rare with higher risk associated with osteoporosis, rheumatoid arthritis, advanced age, female gender, chronic steroid use, metabolic bone disorders, PS type of femoral implant and difficult exposure of the joint due to severe deformities. Patients with risk factors warrant careful pre-operative planning with a full medical history, evaluation of bone stock and availability of appropriate instruments and implants. Exposure, bone preparation and trialing, final femoral implant placement, preparation for the tibial keel and impaction of the tibial component are associated with higher incidence of these fractures. A plethora of management options have been utilized according to surgeon preference. Standard principles of fracture fixation and arthroplasty principles should be followed to achieve stable internal fixation and any unstable fracture site should be bypassed with the utilization of stemmed components. Satisfactory radiographic and functional outcome can be expected with appropriate treatment.

## Data Availability

Data provided in the article.
